# Compaction Behavior of Co-Amorphous Systems

**DOI:** 10.3390/pharmaceutics15030858

**Published:** 2023-03-06

**Authors:** Cecilie-Mathilde Sørensen, Jukka Rantanen, Holger Grohganz

**Affiliations:** Department of Pharmacy, University of Copenhagen, DK-2100 Copenhagen, Denmark

**Keywords:** co-amorphous, tablet, compactability, stability

## Abstract

Co-amorphous systems have been shown to be a promising strategy to address the poor water solubility of many drug candidates. However, little is known about the effect of downstream processing-induced stress on these systems. The aim of this study is to investigate the compaction properties of co-amorphous materials and their solid-state stability upon compaction. Model systems of co-amorphous materials consisting of carvedilol and the two co-formers aspartic acid and tryptophan were produced via spray drying. The solid state of matter was characterized using XRPD, DSC, and SEM. Co-amorphous tablets were produced with a compaction simulator, using varying amounts of MCC in the range of 24 to 95.5% (*w*/*w*) as a filler, and showed high compressibility. Higher contents of co-amorphous material led to an increase in the disintegration time; however, the tensile strength remained rather constant at around 3.8 MPa. No indication of recrystallization of the co-amorphous systems was observed. This study found that co-amorphous systems are able to deform plastically under pressure and form mechanically stable tablets.

## 1. Introduction

Poor aqueous solubility is recognized as a major challenge for the oral administration of drugs, as solubility and permeation are two mechanisms that can impact the bioavailability of a given drug. One way to improve the fate of poorly water-soluble drugs is to convert the crystalline active pharmaceutical ingredient (API) into an amorphous form [1,2]. The downside of this conversion is that amorphous forms are defined as high-energy variations of a compound, and, therefore, these systems are thermodynamically driven to move to a lower energy state, i.e., to recrystallize. In order to stabilize this inherently unstable amorphous form kinetically, various approaches such as polymer-based amorphous solid dispersions (ASDs), mesoporous systems, and co-amorphous systems have been introduced [3,4,5,6,7,8]. Co-amorphous systems are defined as multicomponent amorphous single-phase systems and are combinations of an API with, usually one, low-molecular-weight compound. Initially, drug–drug combinations were investigated; however, as this required not only physical compatibility but also pharmacological relevance, excipients were soon seen as a more universal platform. The most commonly investigated excipients are amino acids; this is due to their natural occurrence and the large variations in the physical properties of their side chains. Co-amorphous systems are most commonly produced via spray-drying or via mechanical activation. Co-amorphous forms are stabilized through salt formation, hydrogen bonds, π-bonds, intimate mixing, or through anti-plasticizing effects; thus, choosing a proper co-former is critical to achieving optimal physical stabilization [5,9,10,11].

As the co-amorphous approach is mostly applied to drugs with originally poor solubility upon oral administration, formulation as a tablet is a rational approach [12]. Direct compression, being the most preferred production method, is the tableting of a blended mixture of ingredients without any additional preliminary processes. Therefore, direct compression is dependent on the particulate properties of the components. The powder mixture should be able to be compressed and compacted into a robust tablet [13], and this powder mixture should have sufficient flowability for the tableting process. Depending on the intended dose of the API, the filler often forms a major part of a tablet formulation and can thereby determine the compressibility and compactability of the tablet as a whole [14,15]. However, for high-dose formulations, the contribution of the API to the plastic and elastic behavior cannot be neglected [16].

It also has to be considered that the downstream processing of an amorphous material into a final dosage form, such as a tablet, can be associated with various kinds of stress, such as moisture, mechanical stress, or thermal stress. In the tableting process, especially, the applied mechanical stress needs to be considered with regard to affecting the system. The question arises of whether mechanical stress will influence the amorphous solid form of the system. Recrystallization of the amorphous material was earlier reported to occur and, as a consequence, influence the stability and dissolution behavior of the API [17].

Alongside lipid-based formulations, amorphous solid dispersions (ASDs) are a common approach to improve the solubility of poorly water-soluble drugs. ASDs are molecular blends of a drug and a polymer, often requiring a relatively high amount of polymer. In the downstream processing of ASDs toward tablets, some challenges have been reported [17,18]. Due to the high polymer-to-drug ratio, a high amount of ASD powder is needed when formulating high-dose tablets, resulting in tablets with inappropriate sizes [18]. This is especially a challenge when a high dose of a drug, such as itraconazole, with limited miscibility in the polymer is reported to require an even larger amount of polymer, leading to an undesirably large tablet [17]. ASD-based tablet formulations of itraconazole, moreover, show poor compressibility and compactability [19]. Further literature reported that the drug loading in ASDs tablet formulations influenced the compaction properties, including tensile strength, disintegration behavior, and the risk of compaction-induced crystallization [20,21,22]. The tensile strength was negatively affected by increasing amounts of API. The presence of amounts as low as 5% of acetaminophen already led to a sharp decrease in the tensile strength, and a further decrease in tensile strength was found in the range of 5–15%, due to a reduced particle bonding strength [20,23]. ASD-based tablets were also found to display prolonged disintegrating times due to the polymer gelation effect [17,24]. Finally, phase separation or crystallization was observed when formulating high (>50%) doses of ASDs as tablets [23].

In this study, the amino acids L-aspartic acid (ASP) and L-tryptophan (TRP) were used as low-molecular-weight excipients and co-formers in a co-amorphous drug formulation with the poorly water-soluble BCS class II drug carvedilol (CAR). It was the aim of this study to investigate the compaction properties and the solid form stability of co-amorphous systems. Due to the small-scale production facilities for the co-amorphous formulations, the investigation of properties that would require larger sample amounts, such as flowability and friability, was not conducted in the current state. The two different co-amorphous systems were chosen as well as investigated as representatives of a salt-forming co-amorphous system (CAR:ASP) and a non-salt-forming system (CAR:TRP). This decision was made to investigate whether the molecular interactions in the co-amorphous systems would influence tablet properties as a whole. This study aims to investigate the influence of the type and content of co-amorphous material on the compaction properties and the solid-state form of the obtained compacts. Promising compaction properties would then allow for more versatile studies on the compaction of co-amorphous systems, including aspects such as flowability, dissolution, long-term physical stability, and the interplay with the various tableting excipients.

## 2. Materials and Methods

### 2.1. Materials

Carvedilol polymorphic form II (CAR, M=406.47 g/mol, code GIVJUQ01) was obtained from Hovione FarmaCiencia (Loures, Portugal) (GIVJUQ01). Carvedilol exhibits pH-dependent solubility with a pKa of 7.8 and is practically insoluble at pH values higher than 9.0. The solubility of carvedilol is around 23 μg/mL at a pH of 7. The glass transition temperature of amorphous CAR was reported at around 40 °C [25]. L-aspartic acid (ASP, M=133.11 g/mol) was purchased from Calbiochem-novabiochem AG (Darmstadt, Germany), L-tryptophan (TRP, M=204.23 g/mol) was purchased from Sigma Aldrich (St. Louis, MO, USA), and microcrystalline cellulose, grade Avicel 102, (MCC, M=370.35 g/mol) was purchased from Fagron Services B.V. (DB Uitgeest, The Netherlands). All substances were of reagent grade. Ethanol of 96% and demineralized water were used as solvents.

### 2.2. Methods

#### 2.2.1. Preparation of Co-Amorphous Material

A Büchi B-290 spray drier equipped with an inert loop B-295, a dehumidifier B-296 (BÜCHI Labortechnik AG, Flawil, Switzerland), and a two-fluid nozzle (diameter 0.7 mm) was used to prepare the co-amorphous material. The drying airflow rate was set to ~40 m^3^/h and the atomizing air flow rate was 667 L/h.

Co-amorphous formulations with a molar ratio of 1:1.5 of CAR:ASP and 1:2.3 of CAR:TRP in 50% ethanol/water were selected. These ratios were judged as the optimal molar ratios for the respective systems in earlier studies [26,27]. Solutions with a total concentration of 2.4 mg/mL of CAR:ASP and 1.1 mg/mL of CAR:TRP, respectively, were prepared, based on the methodology of the earlier cited studies and solubility studies. The applied concentrations were chosen as the highest obtainable concentrations without the application of an ultrasonic bath. CAR was dissolved in ethanol, and the amino acid was dissolved in water. Solubilization was promoted via sonification, whereafter the two solutions were mixed. This resulted in the formation of a clear solution. In order to ensure that the processing conditions would support the formation of a co-amorphous system in the glassy state, the spray-drying conditions were adjusted to reach an outlet temperature below the T_g_ of the respective systems. These temperatures were earlier reported to be 83.7 ± 1.2 °C and around 95 °C for CAR:ASP and CAR:TRP, respectively [26,27]. Solutions of CAR:ASP were spray-dried using an inlet temperature of 100 °C and a feed rate of 6 mL/min, resulting in an outlet temperature of 49 ± 1 °C. Solutions of CAR:TRP were spray-dried using an inlet temperature of 95 °C and a feed rate of 7.5 mL/min, resulting in an outlet temperature of 40 ± 1 °C. All freshly prepared dry powder samples were collected from the cyclone and the collecting vessel and subsequently stored in a desiccator (0% RH, ~25 °C) before further handling and analysis.

#### 2.2.2. Solid-State Characterization

The solid state was characterized using an X’Pert PANalytical PRO X-ray diffractometer (Almelo, The Netherlands), using CuK_α_ radiation (1.54187 Å) and an acceleration voltage and current of 45 kV and 40 mA, respectively. Samples were placed on a clear aluminum sample holder and scanned from 5 to 30° 2θ in reflection mode with a step size of 0.0260° 2θ. Data were collected and analyzed using X’Pert Data Collector software (Malvern Panalytical Ltd., Malvern, UK).

Thermal characterization of the samples was obtained using Discovery DSC (TA Instruments, New Castle, DE, USA). According to the type of sample, around 1.5–3.6 mg of each powder sample was weighed into aluminum hermetic pans and sealed with lids with pinholes. The samples were equilibrated at −20 °C for 5 min and then heated up to 200 °C with a heating rate of 10 °C/min. DSC analysis was conducted under a nitrogen purge at a flow rate of 50 mL/min. Trios software (TA Instruments, New Castle, DE, USA) was used to determine the glass transition temperature, T_g_.

#### 2.2.3. Morphology of Co-Amorphous Material

The morphology of the samples was investigated using scanning electron microscopy (TM3030, Hitachi High-Tech Europe GmbH, Krefeld, Germany) at an accelerating voltage of 15 kV. The samples were mounted on aluminum stubs with double-sided carbon adhesive tapes and coated under vacuum with gold in an argon atmosphere with a Sputter Coater 108auto (Cressington Scientific Instruments Ltd., Watford, UK).

#### 2.2.4. Pycnometric Particle Density

The pycnometric particle density was determined for each mixture with an AccuPyc 1330 helium pycnometer (Micromeritics, Norcross, GA, USA). The samples were measured by adding 1–2 g of the co-amorphous material into the chamber, followed by the addition of MCC to simulate the different compositions (%, *w*/*w*) of the tablet formulations. The pycnometric particle density was used to measure the relative density of the compact, which was then used for the determination of the compressibility of the systems via a Heckel plot.

#### 2.2.5. Compaction Study

The compositions of the different mixtures are shown in Table 1, also indicating the actual contents of CAR. These portions were chosen to provide a wide and realistic span of the investigated API content. Taking into account the various uses of CAR, where doses vary from 3 mg up to 50 mg, the investigated contents are of practical relevance and give an indication of the behavior of both low- and high-dose formulations. The corresponding amounts of co-amorphous material and MCC were weighed into a 50 mL Eppendorf centrifuge tube and blended 3 times for 30 s each using an orbital shaker (Vortex 1, IKA^®^-Werke GmbH & Co. KG, Staufen, Germany).

Of each powder blend, 6 tablets with a weight of 100–105 mg were produced. The compaction of the co-amorphous tablet formulations was performed using a compaction simulator (Gamlen Tableting Ltd., Nottingham, UK) with a 6 mm diameter flat-faced punch and a 500 kg load cell. The compaction settings were fixed at a speed of 60 mm/min and a target load of 225.0 kg. Magnesium stearate in acetone, used as an antiadhesive agent, was applied onto the die and punch before each compaction. During compaction, position (mm) and load (kg) were recorded, and the maximum compression force was determined. From each tablet, force–displacement curves were recorded, and Prism 9 (GraphPad Software, LLC, San Diego, CA, USA) was used to measure the distribution of plastic and elastic work. The percentage of elastic work was calculated based on the ratio between the elastic region and the overall area under the curve (total work).

#### 2.2.6. Disintegration of Co-Amorphous Tablets

Disintegration was performed on three tablets from each formulation using a DT50 Sotax disintegration apparatus (Sotax AG, Basel, Switzerland), and as described in Ph. Eur 2.9.1. The disintegration of tablets and capsules was performed using a volume of 800 mL of water in a 1000 mL vessel. The temperature of the water bath was 37 ℃, and the instrument was equipped with automatic endpoint detection for complete disintegration. The disintegration test was automatically stopped after 15 min.

## 3. Results and Discussion

### 3.1. Characterization of Co-Amorphous Material

**Figure 1:**
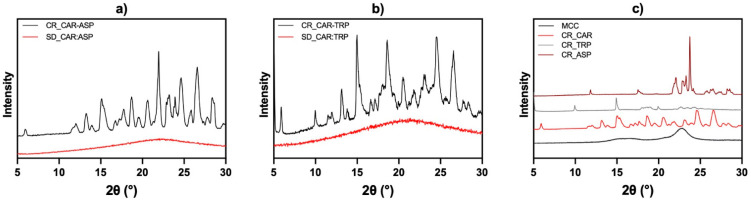
XRPD of physical mixture (CR) and spray-dried system (SD) of (**a**) CAR:ASP and (**b**) CAR:TRP, and (**c**) XRPD of crystalline MCC, CAR, TRP, and ASP.

The first step in the evaluation of a co-amorphous system is to ensure that an actually amorphous system was obtained, followed by an investigation of whether this system is indeed a homogenous one-phase system. This is frequently carried out via XRPD analysis with subsequent analysis via DSC. Diffractograms of the crystalline starting materials, the physical mixtures of the crystalline components, and the obtained material upon spray-drying are shown in Figure 1. The diffractograms of the crystalline starting materials and of the physical mixtures show sharp reflections, proving the crystalline nature of the sample. In contrast, the spray-dried materials did not show any such reflections, but the characteristic halo indicated the amorphous nature of the sample. mDSC measurements of the spray-dried systems showed a single T_g_ for both systems, indicating the formation of a single phase and thus a co-amorphous system. The T_g_ values were found to be 72.6 ± 1.7 °C for CAR:ASP and 79.6 ± 1.7 °C for CAR:TRP (the data are not shown), wherein the glass transition temperature of crystalline CAR was found to be around 40 °C [27]. The glass transition temperature values of the two co-amorphous systems are lower than earlier reported values but are in the same area, and the variation can be attributed to a different production process, including different storage times and residual moisture. In any case, the found T_g_s were far above the outlet temperature of the spray-drying process, ensuring that the product would be in a glassy state. Earlier comparisons of experimental T_g_ values and theoretical T_g_ values derived from the Gordon–Taylor equation have shown that co-amorphous CAR:TRP follows the Gordon–Taylor equation. This means that no strong molecular interactions exist in this system. On the other hand, co-amorphous CAR:ASP positively deviated from the Gordon–Taylor equation, indicating a strong molecular interaction. This interaction has been proven to be in the formation of a co-amorphous salt between the basic functionality of CAR and the acidic functionality of the ASP side chain [26,27].

**Figure 2:**
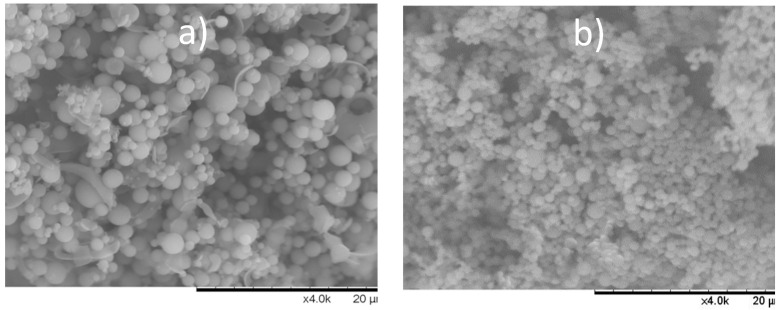
SEM of (**a**) CAR:ASP ×4000 and (**b**) CAR:TRP ×4000.

The morphology of the spray-dried material was investigated using SEM and is shown in Figure 2. The appearance of the spray-dried amorphous material is dominantly one of agglomerated clusters of spherical particles. The particle sizes are judged to be similar, in the range of 1–5 µm with CAR:ASP particles and 0.5–2.5 µm with CAR:TRP particles.

### 3.2. Compaction Behavior of Co-Amorphous Systems

Following the successful production and characterization of the co-amorphous systems, the compaction behavior of the two systems was investigated. The interplay between the MCC-to-co-amorphous-substance ratio was investigated with regard to compressibility and compactability.

#### 3.2.1. Heckel Model

**Figure 3:**
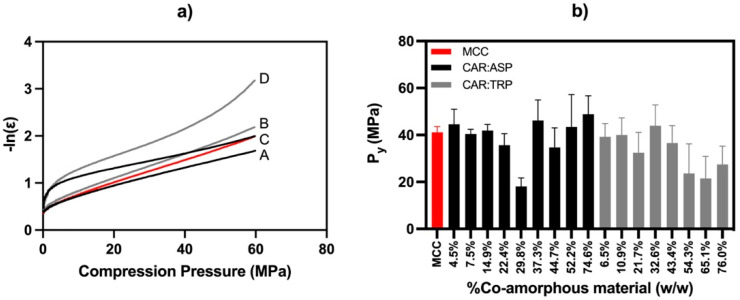
(**a**) Heckel model of 4.5% (*w*/*w*) CAR:ASP (A), 6.5% (*w*/*w*) CAR:TRP (B), 74.6% (*w*/*w*) CAR:ASP (C), 76.0% (*w*/*w*) CAR:TRP (D), and MCC (red line); (**b**) yield pressure derived from Heckel model as bar charts of MCC (red), CAR:ASP (black), and CAR:TRP (grey); n = 6, average ± st. dev.

The compressibility of the tablet formulations was investigated using the “in die” method of the Heckel model, with the porosity being calculated after determination of the pycnometric particle density for each composition. The results are depicted in Figure 3a. Although it has been shown that great differences between published Heckel parameters can be observed from various studies, for example, based on the tablet dimensions, the maximum applied pressure, or the applied method (”in die” versus “out of die”) [28], they can still provide a suitable ground for comparing similar blends at constant conditions. It has to be noted that, in the current study, the applied maximum pressure via the compaction simulator of around 80 MPa was rather low compared with other studies in which pressures of up to 500 MPa were investigated [29]. The Heckel model assumes that pore volume is reduced during compression and follows a first-order process, whereby a relationship between the relative porosity of the tablet powder and the applied pressure can be found. A linear correlation was observed for all investigated samples, usually in the range 20–60 MPa. The yield pressures (1/slope) of the fitted linear regression lines are depicted in Figure 3b. A small yield pressure indicates a faster onset of plastic deformation at low pressures and therefore good compressibility [30,31].

The yield pressure_,_ P_y_, of the pure MCC tablets was calculated using the Heckel model and was found to be 41 ± 2 MPa. When looking at the influence of increasing ratios of the co-amorphous material, on a general level, rather similar values were found throughout this study, ranging from 18 to 49 MPa. Inside this rather homogeneous field of observations, one tendency can be observed for CAR:TRP systems. Herein, a slight decrease in yield pressure at high CAR:TRP portions may occur, indicating better compressibility with increasing portions of co-amorphous material. In general, all tablets displayed yield pressures in a similar range to pure MCC tablets. This confirms the ability of co-amorphous tablets to be plastically deformed under pressure.

#### 3.2.2. Elasticity of Co-Amorphous Material

**Figure 4:**
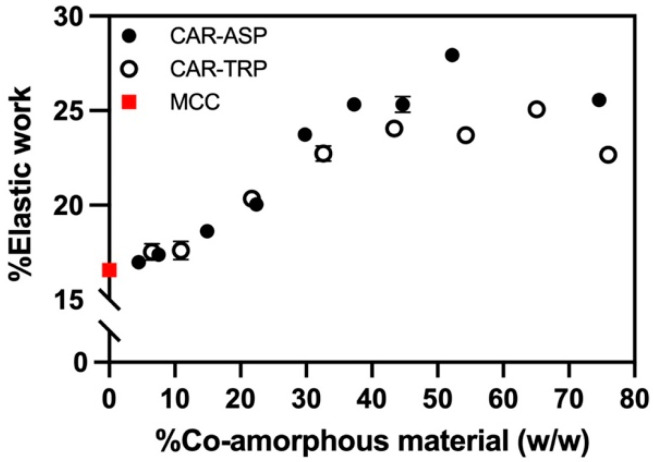
Elastic work (%) of CAR:ASP (%, *w*/*w*) (closed circle), CAR:TRP (%, *w*/*w*) (open circle), and MCC (red square) tablets calculated. n = 6, average ± st. dev.

Force–distance curves were obtained for all compositions. Based on these force–displacement curves, a distribution between elastic and plastic work could be established. The elastic work was calculated as a percentage of the total work from the force–distance curves and is shown in Figure 4. For comparison, a total of 6 MCC tablets showed an elastic work of 16.6 ± 0.3%.

Figure 4 depicts the changes in the relative contribution of the elastic work with increasing CAR–amino acids ratios. For both types of co-amorphous systems, an increase in co-amorphous material led to an increase in elastic work, up to compositions with around 35% of co-amorphous material, wherein a plateau in the percentage of elastic work was reached. Despite this increase in elasticity, the majority of the applied work is still converted into plastic deformation, thereby enabling the production of tablets.

### 3.3. Properties of Co-Amorphous Tablets

**Figure 5:**
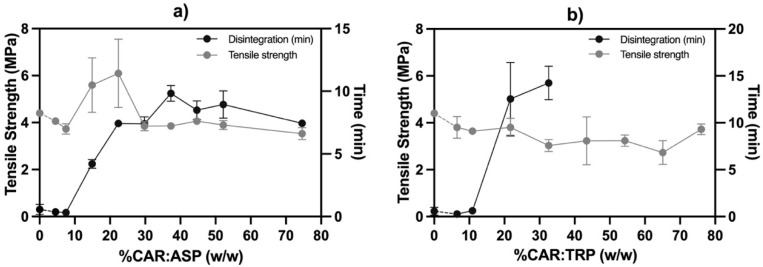
Tensile strength and disintegration of (**a**) CAR:ASP + MCC tablets (%, *w*/*w*) and (**b**) CAR:TRP + MCC tablets (%, *w*/*w*). n = 3, average ± st. dev.

Following compaction, the properties of the obtained tablets were investigated with regard to their tensile strength and their disintegration behavior (Figure 5). For both systems, the tensile strength was rather constant when varying the content of co-amorphous material in the broad range of 5–75%. No obvious difference in evolution could be observed between CAR:ASP and CAR:TRP. Furthermore, the absolute values of the tensile strength for both systems were rather similar, i.e., around 3.8 MPa. Although an initial increase in tensile strength up to around 20% CAR:ASP seems to appear, the deviation is not statistically significant due to a high standard deviation at these measurement points. Furthermore, the observed difference in the particle sizes of the co-amorphous systems did not affect the tensile strength. It is therefore concluded that neither the different types of co-amorphous systems nor the increasing contents of the co-amorphous material in the tablets significantly influenced the tensile strength. The suitability of the co-amorphous systems to be compacted can thus be assumed. It should be noted that the flowability of the co-amorphous material was not investigated at this stage due to the limited amounts of co-amorphous material available. Flowability remains an important consideration for future studies, especially when considering larger-scale production.

However, with regard to disintegration behavior, several differences could be observed. As stated, a comparable tensile strength was achieved for all tablets. Nevertheless, tablets with a content of co-amorphous material of up to around 10% disintegrated very fast in less than a minute, showing the same behavior as pure MCC tablets [15]. At higher contents of co-amorphous material, an increase in the disintegration time was seen.

For the CAR:ASP systems, the disintegration time reached around 8 min with a CAR:ASP content of 20% and from there, increased slightly towards a plateau at around 10 min. The disintegration of the CAR:ASP tablets complies with Ph. Eur 2.9.1 of complete disintegration of uncoated tablets within 15 min. The fast disintegration performance of the CAR:ASP tablets might be attributed to salt formation between CAR and ASP, as salt formation is a well-known approach for increasing the solubility and dissolution rate of poorly water-soluble drugs [26,27,32].

In contrast, the disintegration time increased sharply for the CAR:TRP tablets and approached 15 min, already at around 30% of CAR:TRP. At higher CAR:TRP contents, the tablets did not disintegrate within the expected time of 15 min. It should be noted that the tablets in this study were not optimized in regard to disintegration and that the observed challenge with disintegration can potentially be solved with the addition of a super-disintegrant. For non-salt-forming co-amorphous systems, it might, therefore, be a necessity to add a disintegrant to the composition, while the disintegrant might be omitted for salt-forming co-amorphous systems. In any case, it can be seen that the type of co-amorphous material clearly influences the disintegration time of the tablets as a whole and becomes dominant when there is above 20% of the co-amorphous material in the tablet. The percolation threshold is usually found in this range, and the current observation is in good agreement with a recent study that reported a step-change change in dissolution and disintegration behavior upon passing the percolation threshold [33]. In the current study, the salt-forming co-amorphous system still enabled disintegration, while the non-salt-forming system prevented disintegration. Due to the focus of the current study on the compaction behavior, and the detailed investigation of the dissolution of both co-amorphous systems in earlier work, dissolution experiments were not performed. Previous studies on the dissolution behavior of the two co-amorphous powders showed an improved dissolution behavior compared with crystalline CAR. Both CAR:ASP (1:1.5 molar ratio) and CAR:TRP (1:1 molar ratio) showed a faster dissolution rate that reached a degree of supersaturation of around three, as well as maintenance of the supersaturation to a certain degree, resulting in an area under the dissolution curve of around 2.5 times the area of crystalline CAR [34,35]. For CAR:ASP tablets, a similar behavior of the compacts would be expected, whilst the non-disintegrating CAR:TRP systems would require the addition of a disintegrant.

### 3.4. Compaction-Induced Crystallization of Co-Amorphous Tablets

**Figure 6:**
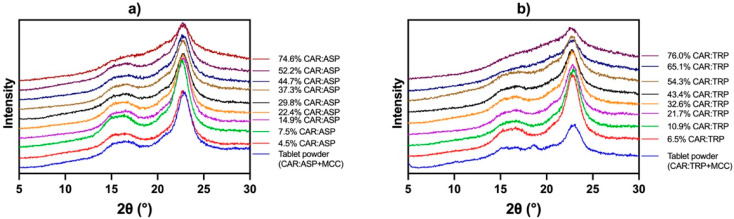
X-ray powder diffractograms of crushed (**a**) CAR:ASP tablet formulations (%, *w*/*w*) and powder mixture and (**b**) CAR:TRP tablet formulations (%, *w*/*w*) and powder mixture. Intensity off-set to enhance comparability.

Applying mechanical forces, such as compression, may induce phase separation and nucleation and hence facilitate the recrystallization of the co-amorphous tablets, namely, “compression-induced crystallization” [36]. In order to investigate whether this phenomenon occurred in the current systems, a tablet from each formulation was gently crushed with a pestle. The solid states of the obtained powders were investigated using XRPD. Figure 6 shows the diffractograms of the crushed tablet powders as well as the diffractograms of a reference powder mixture of a spray-dried co-amorphous material prior to compaction. A characteristic reflection related to the crystalline part of MCC is seen at 22–23° 2θ.

Besides the reflection originating from MCC, Figure 6 shows no crystalline reflections in either the CAR:ASP or CAR:TRP tablets. The reflection originating from MCC can be seen in all systems, and a decline in this signal (due to a lower proportion of MCC in the system) can be seen. However, no additional reflection of either CAR or any of the amino acids was observed upon compaction. Comparing the diffractograms of the tablet powder before compaction with those of the crushed tablet powder, it can be assumed that no compaction-induced crystallization occurred at an applied pressure of up to 80 MPa. It can thus be concluded that an applied mechanical force will not affect the co-amorphous nature of the systems and that compaction of co-amorphous systems can be deemed feasible. It remains to be investigated whether this property would also be seen at higher compaction pressures, which would often be applied in an actual tablet press. Considering that hard tablets with a tensile strength of around 4 MPa were obtained already at the currently applied pressure, a strong deformation of the powder particles occurred, without the initiation of recrystallization, indicating a promising system for compaction. Currently, no statements on the physical long-term stability of the compacts can be made. However, considering the high T_g_ of the CAR–amino acid combinations, which is around 50 degrees above room temperature, rapid recrystallization is not expected. Storage stability studies (partly under accelerated conditions) and, for high-dose systems, the increase in flowability of the systems, together with the evaluation of the influence of other excipients such as disintegrants and glidants, would be necessary aspects in further investigations of co-amorphous systems, from compaction behavior to actual tablet production.

## 4. Conclusions

Co-amorphous systems of CAR:ASP and CAR:TRP were compacted with MCC at various ratios ranging from around 5 to around 75% of co-amorphous material. No indications of recrystallization upon compaction were observed; thus, all systems retained their amorphous nature upon compaction. The compressibility and the tensile strength were found to be independent of the type and portion of the co-amorphous material. In contrast, disintegration time increased with increasing contents of amorphous substances. As a major difference, tablets with the salt-forming co-amorphous system still disintegrated within the allotted period, while non-salt-forming systems did not disintegrate. This study shows that the compaction of co-amorphous systems without recrystallization is possible and that the nature of a co-amorphous system can influence the tablet properties.

## Figures and Tables

**Table 1:** Compositions (%, *w*/*w*) of tablet formulations of co-amorphous CAR:amino acid and MCC blend with corresponding CAR percentages.

CAR	CAR:ASP	MCC	CAR	CAR:TRP	MCC
3	4.5	95.5	3	6.5	93.5
5	7.5	92.5	5	10.9	89.1
10	14.9	85.1	10	21.7	78.3
15	22.4	77.6	15	32.6	67.4
20	29.8	70.2	20	43.4	56.6
25	37.3	62.7	25	54.3	45.7
30	44.7	55.3	30	65.1	34.9
35	52.2	47.8	35	76.0	24.0
50	74.6	25.4			

## Data Availability

The data are available on request.
